# Anatomy and Pathology of the Texel Sheep Larynx

**DOI:** 10.3390/vetsci6010021

**Published:** 2019-02-27

**Authors:** Katie Waine, Ben Strugnell, John Remnant, Fiona Lovatt, Martin Green, Hannah Rideout, Elizabeth Genever, Kerstin Baiker

**Affiliations:** 1 School of Veterinary Medicine and Science, University of Nottingham, Leicestershire LE12 5RD, UK; John.remnant@nottingham.ac.uk (J.R.); fiona.lovatt@nottingham.ac.uk (F.L.); martin.green@nottingham.ac.uk (M.G.); kerstin.baiker@nottingham.ac.uk (K.B.); 2 Farm Post Mortems Ltd., Hamsterley House, Hamsterley, Bishop Auckland, Durham DL13 3QF, UK; benstrugnell@aol.com; 3 Animal Production, Welfare and Veterinary Sciences, Harper Adams University, Newport, Shropshire TF10 8NB, UK; rideouth10@gmail.com; 4 Liz Genever Sheep and Beef Consultancy, Bramley Cottage, Uffington, Stamford, Lincolnshire PE9 4SX, UK; liz@lizgenever.com

**Keywords:** texel, bluefaced leicester, sheep, larynx, laryngeal chondritis, texel throat, respiratory disease

## Abstract

Laryngeal chondritis, or “Texel throat”, is a disease affecting the upper respiratory tract of sheep with breeds like the Texel appearing to be predisposed. Previous work suggests the conformation of these breeds of sheep may be predisposing these animals to laryngeal disease. This study evaluated the anatomy of the Texel sheep larynx and describes incidental pathology. Forty-three larynges from rams of the Texel and Bluefaced Leicester breeds of sheep were measured and photographed. A larynx from each breed was submitted for computed tomography (CT) and magnetic resonance imaging (MRI). Measurements, photography, CT, and MRI demonstrated a difference in the anatomy of the larynx between breeds and a higher proportion of Texel sheep had laryngeal lesions. This study supports the hypothesis that the anatomy of the Texel sheep could be pre-disposing the breed to laryngeal chondritis.

## 1. Introduction

Laryngeal chondritis is an upper respiratory tract disease of sheep of unknown aetiology [1,2,3]. The prevalence in the United Kingdom (UK) or on an international level is unknown, but a predisposition of the Texel breed is suggested in the literature [1,2,4] and supported through surveillance data [5]. Laryngeal chondritis has health and welfare implications for the individual sheep affected, as well as consequences for the economics of the farm [3].

The underlying cause of laryngeal chondritis is not clear [3]. Mucosal lesions found at post mortem examination are thought to allow the entry of opportunistic pathogens leading to the formation of abscesses in the laryngeal cartilage [3]. Suggested causes of these mucosal lesions include drenching gun injuries, inhalation of grass awns, and repeated trauma to the larynx during dyspnoea [3,6,7]. The predisposition of the Texel breed to laryngeal chondritis is thought to be due to the short head and neck of the breed affecting the anatomy of the larynx [2], but this has not been proven. Selecting for certain anatomical traits is, however, known to cause inadvertent effects in other domestic animals, such as is seen in brachycephalic obstructive airway syndrome (BOAS) in flat-faced dogs [8].

The Texel is the most commonly used terminal sire in the UK sheep industry, the breed has sired over 12% of the national ewe flock and lambs often receive a premium [9]. No previous studies have assessed the anatomy of the Texel larynx and how this may play a part in predisposing these animals to laryngeal chondritis. This study aimed to assess the anatomy of the Texel larynx and to determine if it could be part of the pathogenesis of laryngeal chondritis.

## 2. Materials and Methods

### 2.1. Ethical Approval

This project received ethical approval (approval number: 2143171026) from the ethics research committee at the School of Veterinary Medicine and Science, University of Nottingham. 

### 2.2. Larynx Selection

Twenty-three larynges were collected from Texel rams, and 20 larynges were collected from Bluefaced Leicester (BFL; used as the control) rams between August 2017 and October 2018. The larynges were labelled TX1–TX23 and BL1–BL20 for the Texel and BFL breeds, respectively. Adult entire males of each breed were selected from fallen stock (animals that have died or been killed on the farm for reasons other than human consumption) brought to a Fallen Stock Collection Centre (a centre for carcase disposal) in Northern England. The characteristic appearances of each breed were used to select carcases for the study, which were collected when time allowed alongside the daily post mortem room workload. Age was estimated by examining the incisor teeth where two tooth (2T) ≈ 12–18 months old, four tooth (4T) ≈ 2 years-old, six tooth (6T) ≈ 3 years-old, full mouth (FM) ≈ 4 years-old and over, and broken mouthed (BM) ≈ older than FM [10]. Farm of origin, clinical history, pedigree, and cause of death were not known, and none of the animals was weighed. The state of post mortem preservation of each whole carcase was assessed at the point of selection with those showing advanced signs of autolysis, such as extreme bloat with loss of wool and extensive green discolouration of the carcase, not chosen for the study. Larynges deemed too autolysed to allow accurate assessment (demonstrated by the collapse of the structure when placed on a table) were also not included in the study.

### 2.3. Carcase Measurements

The length of the head, neck circumference, length of the neck, height and girth of each ram were measured to the nearest half centimetre with the carcase in lateral recumbency using a tape measure (Table 1). The girth measurement was used as a proxy for weight [11].

### 2.4. Larynx Collection, Measuring and Photography

The tongue, larynx and proximal trachea were removed from each carcase using a ventral approach. The tongue and loose soft tissue were removed, and the larynx was placed whole in 10% neutral buffered formalin for at least one week. 

**Figure 1:**
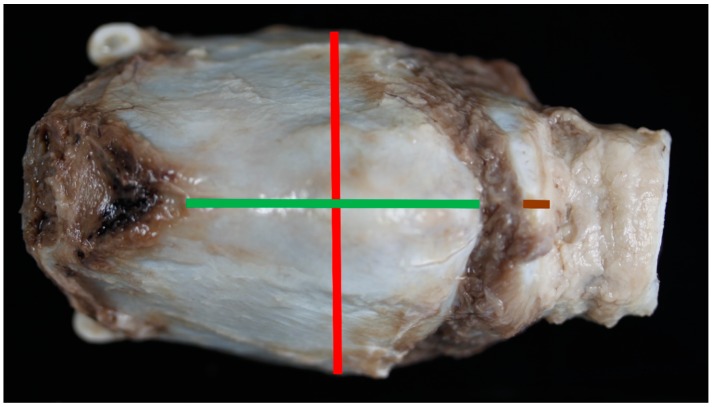
The ventral aspect of the larynx and measurements taken [Green line = measurement #1, red line = measurement #3, brown line = measurement #10].

**Figure 2:**
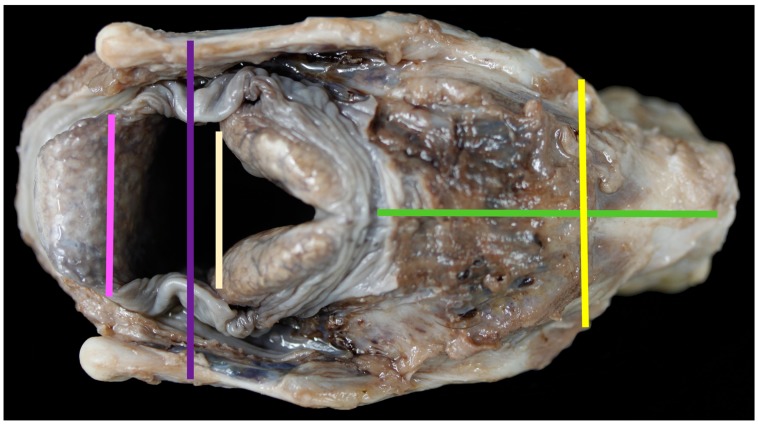
The dorsal aspect of the larynx and measurements taken [pink line = measurement #12; purple line = measurement #4; pale line = measurement #15; yellow line = measurement #5; green line = measurement #9].

**Figure 3:**
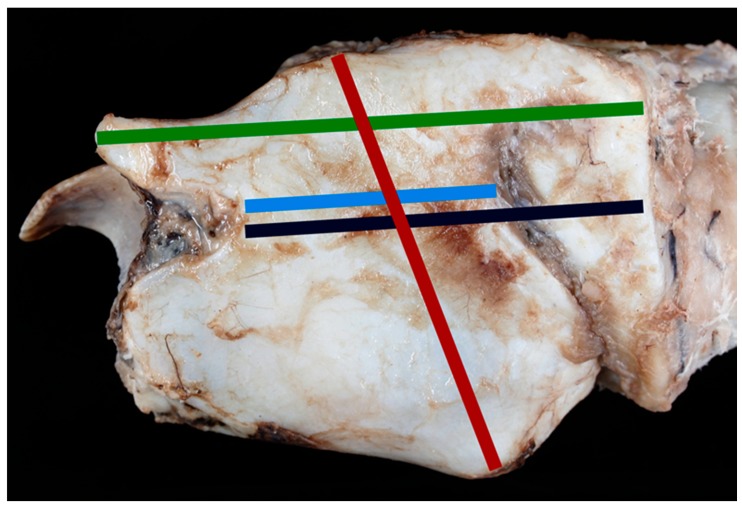
The lateral aspect of the larynx with measurements taken [green line = measurement #2; light blue line = measurement #6; red line = measurement #7, black line = measurement #8].

**Figure 4:**
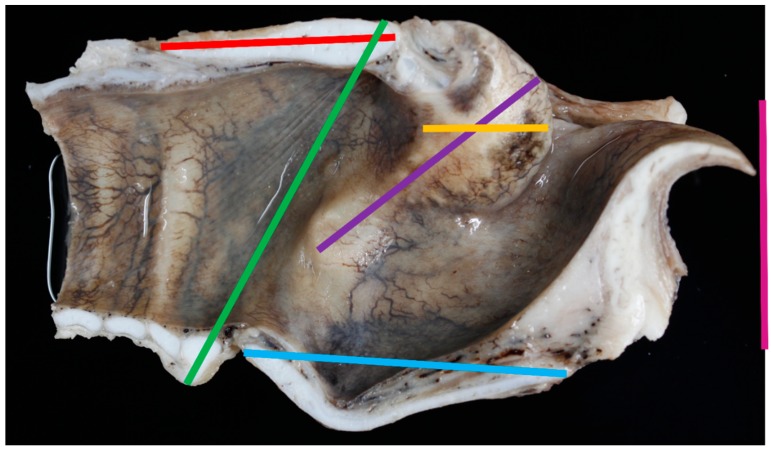
Medial view of the laryngeal lumen with measurements taken [red line = measurement #16; green line = measurement #17; blue line = measurement #18; dark purple line = measurement #19; pink line = measurement #20 (with epiglottis flattened); orange line = measurement #21].

**Figure 5:**
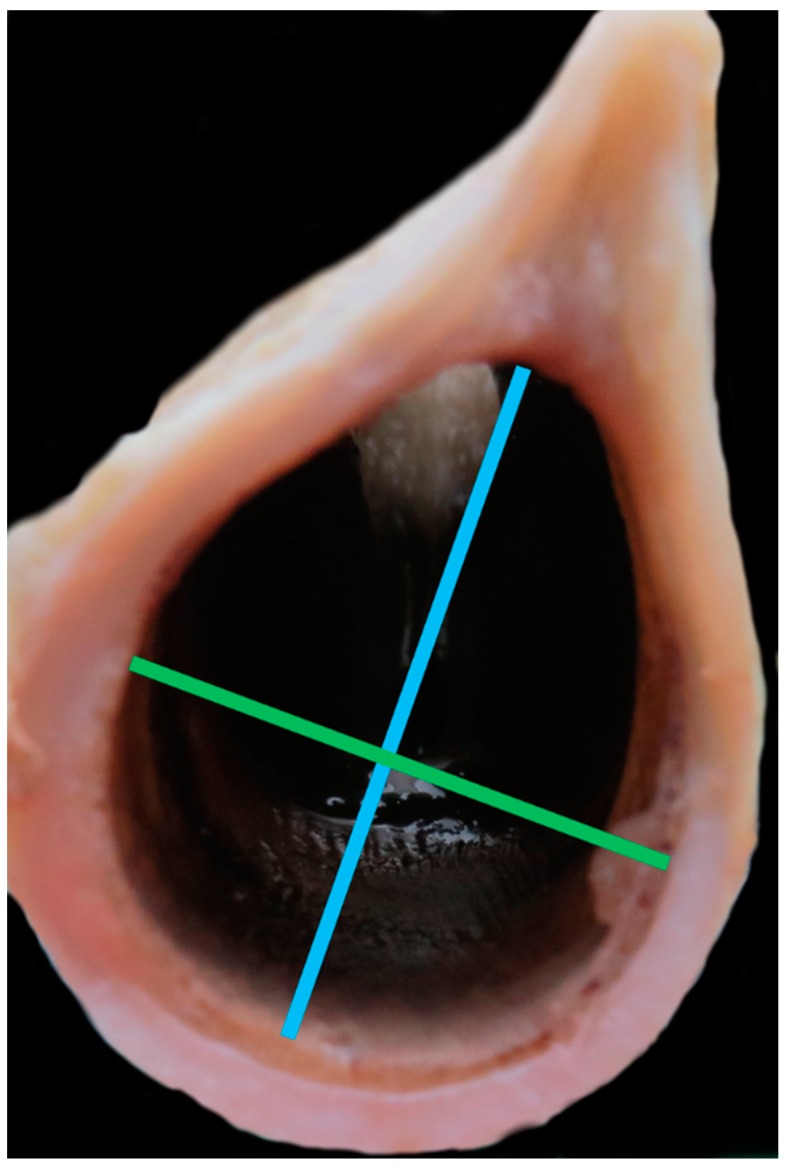
View of the caudal larynx looking cranially with measuring points for the tracheal rings [green line = measurement #13; blue line = measurement #14].

At the University of Nottingham, the fixed larynges were then dissected further to leave the intact larynx with two tracheal rings. Each larynx was photographed using a Canon EOS 1000D (Reigate, UK) digital camera from the dorsal and ventral aspects (Figure 1 and Figure 2), from each side (Figure 3) and the internal medial surfaces (Figure 4). A Canon PowerShot SX50 HS digital camera (Reigate, UK) was used to take photos from the cranial and caudal ends (Figure 5) and an iPhone SE with flash was used to take photos of the laryngeal lumen from the caudal aspect. Larynges were measured and photographed whole, before being split longitudinally with a scalpel blade. Larynges that were mineralised were placed in decalcification solution for 12 to 24 h before splitting. 

**Figure 6:**
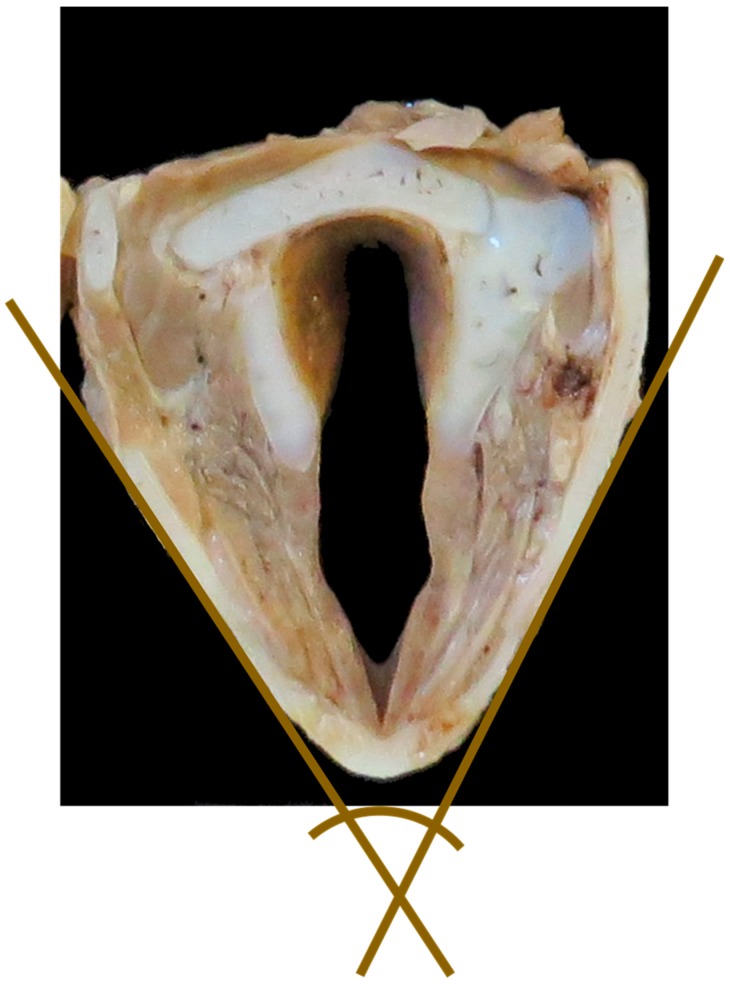
Transverse cross-section of the larynx to show measurement #11, the acute angle between the two brown lines. [NB In the study this measurement was taken with the larynx whole, the transverse section shown here is to demonstrate the angle only].

Measurements of the fixed larynges, based on Zrunek [12], were taken using a pair of compasses, a ruler and a goniometer:

1. Anterior thyroid length (distance from upper to lower thyroid incisure (mm)) (Figure 1)
2. Dorsal thyroid length (distance between tips of upper and lower horns (mm)) (Figure 3)
3. Maximum thyroid breadth (length of thyroid tubercula (mm)) (Figure 1)
4. Upper thyroid breadth (length of thyroid tubercula (mm)) (Figure 2)
5. Lower thyroid breath (out distance of inferior horns (mm)) (Figure 2)
6. Length of thyroid lamina (distance of notches prior to horns (mm)) (Figure 3)
7. Anteroposterior dimension (distance between laryngeal prominence and plane thought posterior margins of laminae (mm)) (Figure 3)
8. Main length of larynx (extension of distance 6 to lower margin of cricoid cartilage (mm))
9. Length of cricoid lamina (mm) (Figure 2)
10. Anterior length of cricoid arch (mm) (Figure 1)
11. Angle between thyroid laminae (°) (Figure 6)
12. Width of epiglottis at attachment of soft tissue (mm) (Figure 2)
13. Width of trachea at second tracheal ring (as felt by palpation) (mm) (Figure 5)
14. Height of trachea at second tracheal ring (as felt by palpation) (mm) (Figure 5)
15. Distance between midpoint cranial corniculate projections (mm) (Figure 2)
16. Split cricoid length (mm) (Figure 4)
17. Cricothyroid distance measuring to outer edges (mm) (Figure 4)
18. Split thyroid length (mm) (Figure 4)
19. Depth of the cranial AC projections (mm) (Figure 4)
20. Flat length of epiglottis (mm) (Figure 4)
21. Height of C-A cartilage (mm) (Figure 4)

The tracheal area was estimated based on the area of an ellipse:

Area of trachea ≈ π × 0.5 tracheal width × 0.5 tracheal height(1)

Each larynx was subjectively assessed for its overall appearance with respect to the shape and the soft tissue surrounding the cartilage at each stage of the study. 

### 2.5. Imaging

Computed tomography (CT) and magnetic resonance imaging (MRI) imaging were carried out to determine if the modalities could be useful for assessing the anatomy and pathology of the fixed sheep larynx. Larynges were sent for imaging before measurements had been taken and while each larynx was intact. Selection was based on the best example of anatomy for each breed from the larynges available at the time (the most intact, with no external lesions and the most cleanly dissected that had not already been incised). One formalin-fixed Texel larynx and one formalin-fixed Bluefaced Leicester larynx were submitted to Scotland’s Rural College (SRUC) CT Scanner Unit, Edinburgh for Computer Tomography (CT) scanning. The larynges were scanned using a 16 Slice Siemens Somatom Scope CT scanner at 0.75 mm slice thickness, 100 mAs, 130 kV, 450mm Field of View and Pitch 1. CT images were viewed using syngo Fast View imaging software (Siemens Healthcare, Erlangen). One larynx from each sheep breed was sent to Bearl Equine Clinic, Northumberland, for MRI. A Hallmarq standing equine distal limb MRI scanner (EQ2 System; Hallmarq Veterinary Imaging Ltd, Guildford, UK) with a low field strength open configuration permanent magnet (0.3 tesla) was used. Three scanning sequences were carried out to ascertain if MRI could produce suitable images to assess laryngeal anatomy and pathology; T1 gradient-echo (GRE), T2 (GRE and fast spin echo (FSE)) and Short-TI Inversion Recovery (STIR). The slice thickness varied from 1.5 mm for the T1 GRE HR (high resolution) images, to 3.5 mm for the T2 FSE and STIR sequences. All MRI images were processed and viewed using eFilm Lite 2.1.0 software (Merge Healthcare, IBM Watson Health, Chicago, IL, USA).

### 2.6. Statistical Analysis

Data from the two breeds of sheep were compared using Pearson’s T-test for continuous variables and Chi-squared or Fischer’s exact test for categorical variables. Basic descriptive statistics were conducted using Microsoft Excel 2016, and the statistical tests were carried out using R V.3.4.0 statistical software [13]. Graphs and tables were made in Excel or R. Statistical significance was set at *p* < 0.01.

## 3. Results

**Figure 7:**
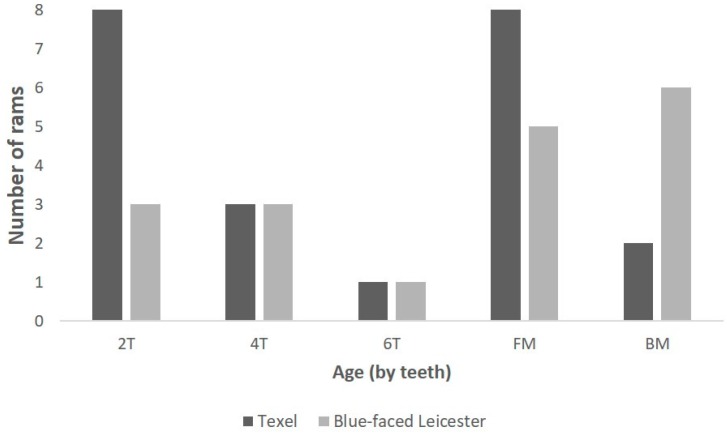
The age of the rams from which the larynges were collected [2T = two-toothed; 4T = four-toothed; 6T = six-toothed; FM = full mouth; BM = broken mouthed].

The age range of the carcases that the larynges were collected from is shown in Figure 7 using the estimated descriptors; there was no significant difference in age category between the breeds of the animals collected.

### 3.1. Anatomy of the Larynx

#### General Observations

**Figure 8:**
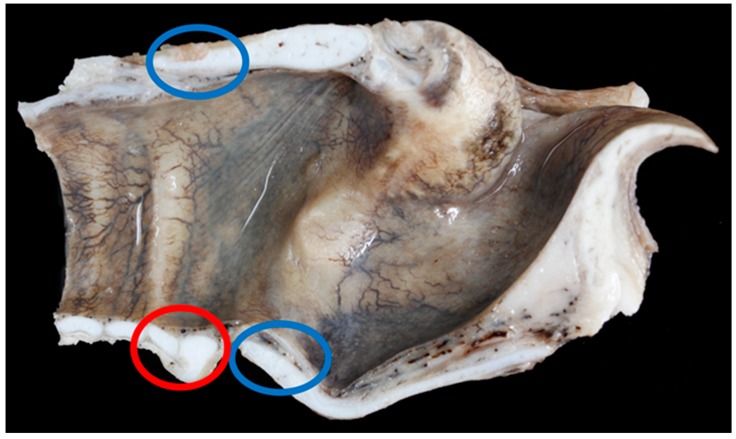
The blue rings show the common places of ossification found in the larynges examined. The red ring shows the cricoid cartilage (right) and the first tracheal ring (left) – in this specimen the tracheal ring is not overriding the cricoid cartilage [BL3].

Nine Texel larynges and eight BFL larynges had points of mineralisation at the caudal upper cricoid and/or the caudal thyroid cartilage that required decalcification (Figure 8). 

**Figure 9:**
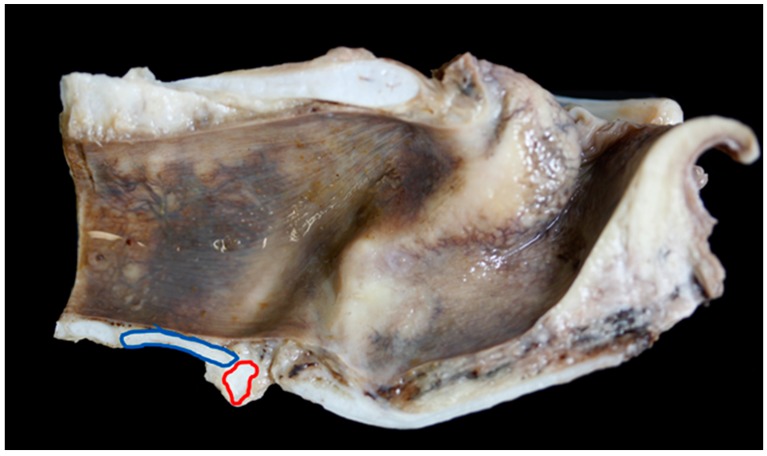
Mild overlap of the cricoid cartilage (red ring) by the first tracheal ring (blue ring) [BL6].

The ventral cricoid ring and the first tracheal ring varied in shape and size, Figure 9 shows a very long and flat tracheal ring in a BFL larynx. 

**Figure 10:**
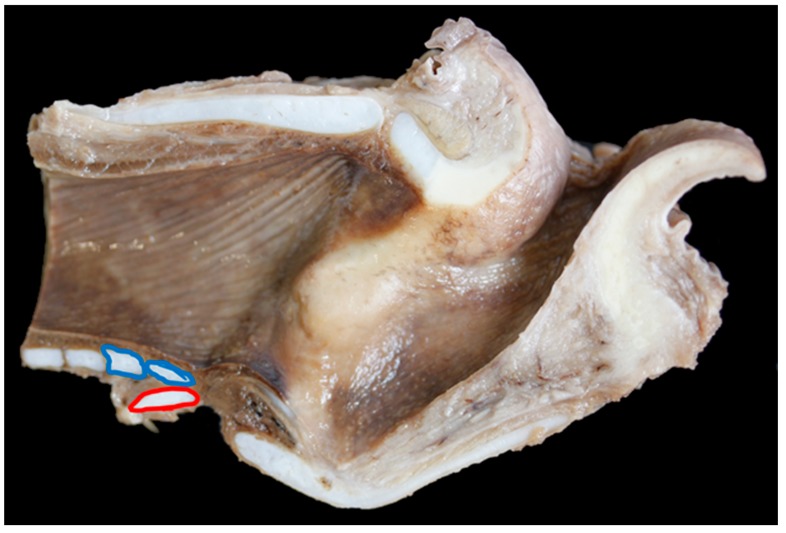
Extreme overlap of the cricoid cartilage (red ring) by the first, and in this case, second tracheal rings (blue rings) [TX5].

In nine of 19 (47%) BFL larynges the first tracheal ring overlapped the cricoid to some degree (an overlap was defined as any part of the first tracheal ring extending cranially beyond the level of the most caudal edge of the cricoid cartilage), in Texel larynges 15 out of 23 (65%) overlapped (Figure 8, Figure 9 and Figure 10). 

**Figure 11:**
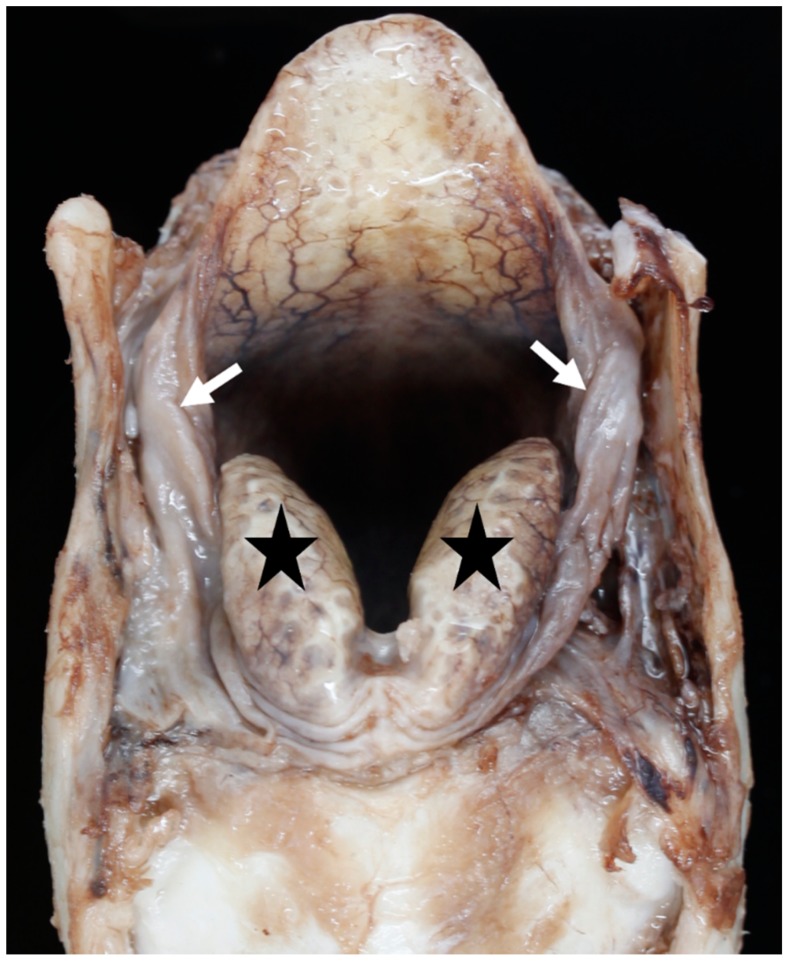
Little excess of soft tissue (arrows) seen around the corniculate processes (black stars) [BL3].

**Figure 12:**
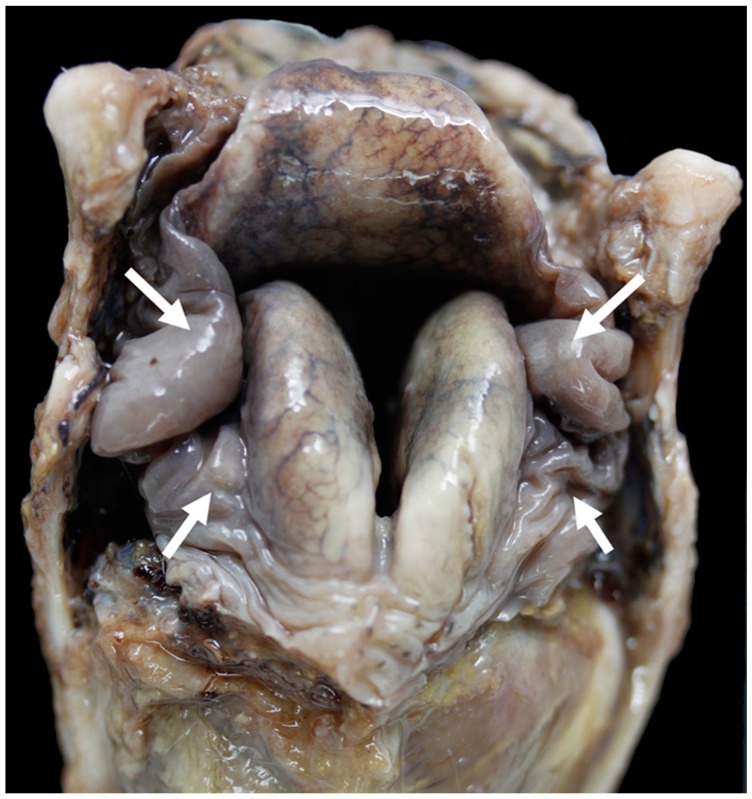
Moderate excess of soft tissue (arrows) around the corniculate processes [BL18].

Overlap of the cricoid by the tracheal rings and varying lengths of the first tracheal ring retrospectively resulted in the tracheal measurements being taken at the level of the 2nd–4th tracheal ring. The amount of soft tissue around the larynx, especially in the space between the epiglottis and corniculate processes, and between the corniculate processes and the thyroid cartilages, varied between individual larynges. Generally, the BFL had very little excess soft tissue (Figure 11), but occasional BFL larynges showed a moderate excess (Figure 12). 

**Figure 13:**
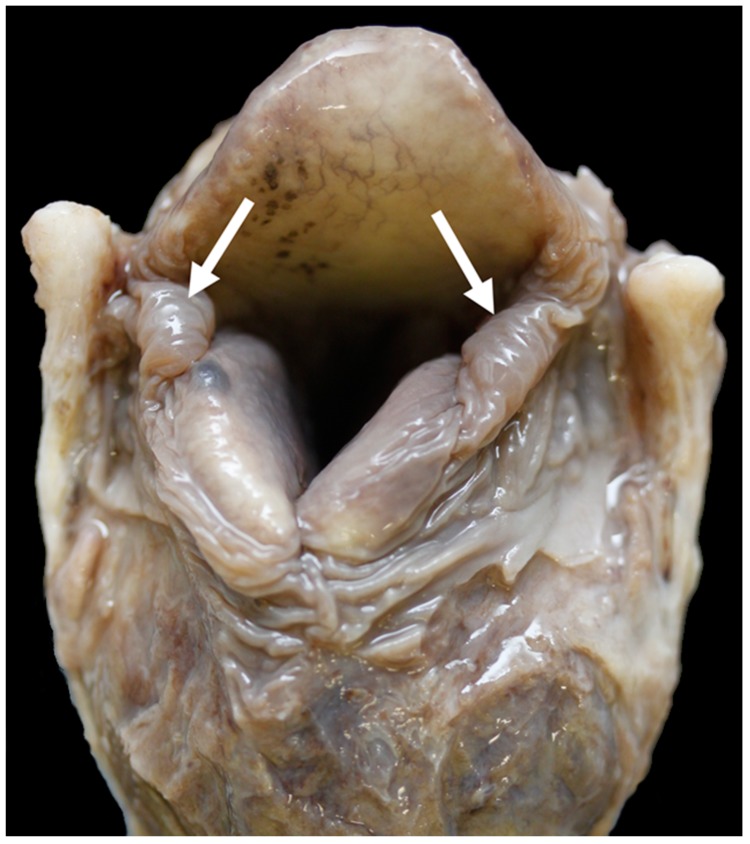
Little excess of soft tissue (arrows) around the corniculate processes [TX9].

**Figure 14:**
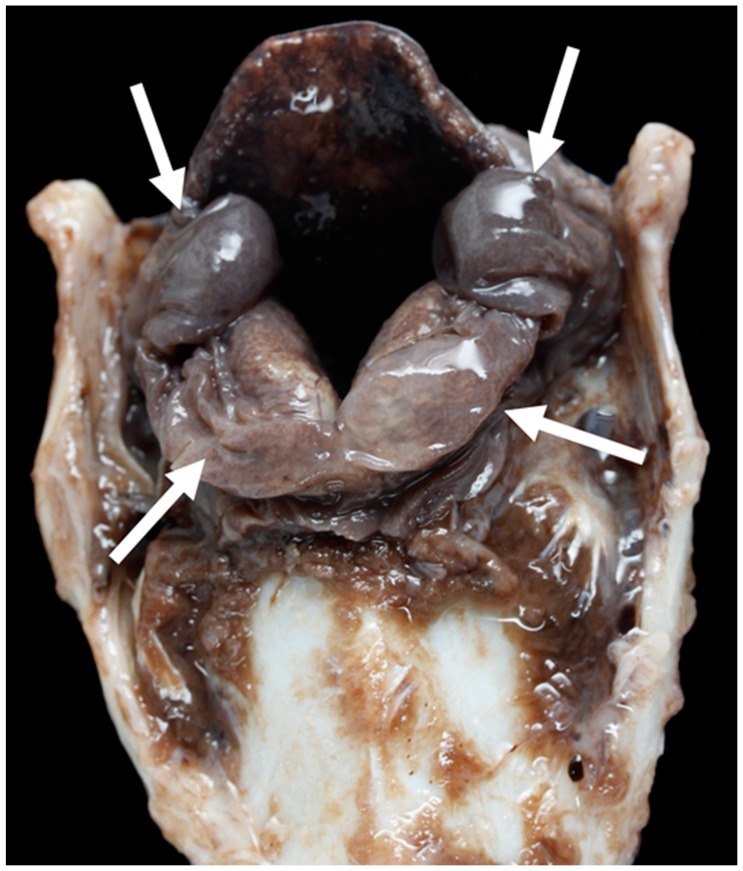
Severe excess of soft tissue (arrows) around the corniculate processes [TX2].

The Texel had mild-severe excess of soft tissue in these spaces (Figure 13 and Figure 14). 

A summary of the measurements is shown in Table 2.

### 3.2. Carcase Measurements

**Figure 15:**
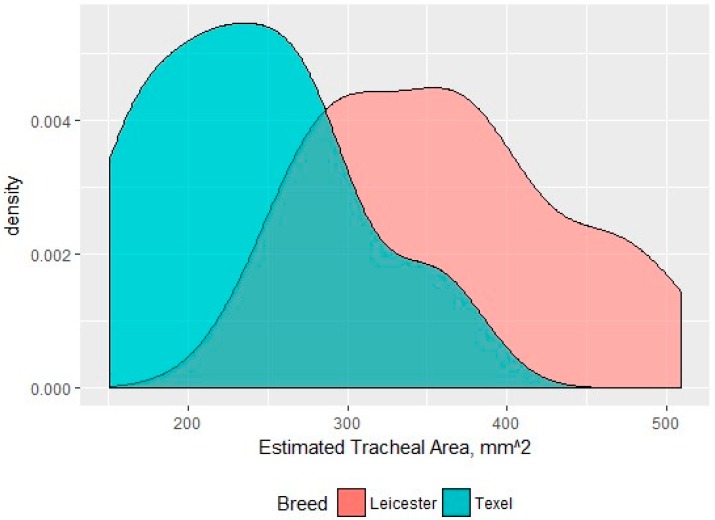
A density distribution of the tracheal areas for each breed measured at the 2nd–4th tracheal ring (mm^2^).

The neck circumference of the Texel rams was significantly larger than that of the Bluefaced Leicester, while the neck length was significantly shorter in the Texel. The Texel sheep were significantly shorter in height than the Bluefaced Leicester, but there was no significant difference in girth measurements between breeds suggesting they had a similar weight range. The area of the trachea at the level of the 2nd–4th tracheal ring of the Texel was significantly reduced in size compared to that of the Blue-faced Leicester (Figure 15).

### 3.3. Larynx Measurements

**Figure 16:**
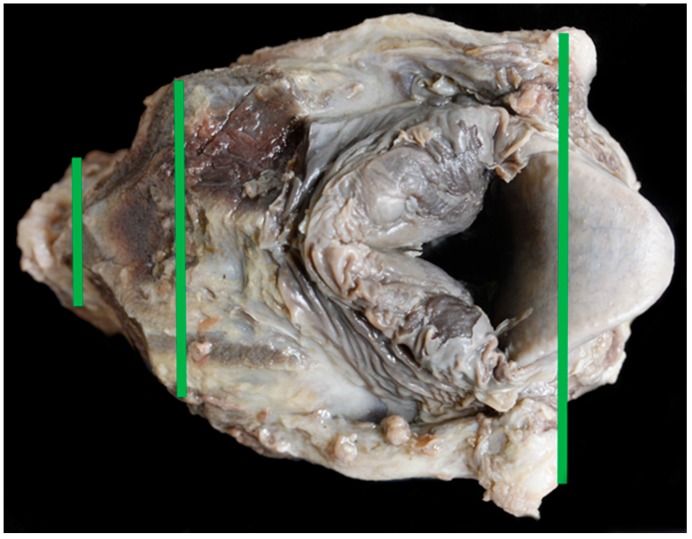
TX11 with a funnel shape to the airway.

**Figure 17:**
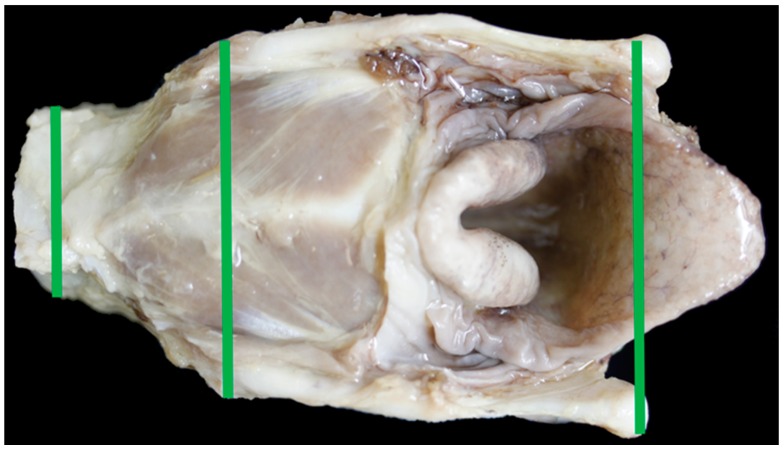
BL9 showing a more elongated larynx.

The Texel larynx was significantly shorter in length and height when compared to the BFL. It was significantly wider at the cranial and mid points than the control and had similar sized arytenoid and epiglottal cartilages. The tracheal width, height and area were significantly smaller in the Texel. There was no relationship between the girth length and the tracheal area in either breed. With a significantly wider cranial end to the larynx, in distance measured and in angle, the Texel larynx narrows to a significantly narrower trachea suggesting a ‘funnel’ shape to the airway (Figure 16 and Figure 17). 

### 3.4. The Glottis

**Figure 18:**
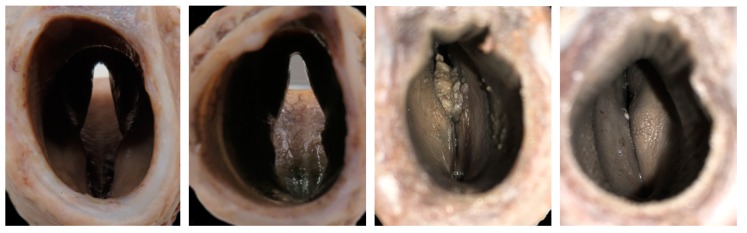
The vocal folds of the BFLs were not found to touch in any of the larynges examined (BL1 and BL3, left two photos). In the Texel sheep (T11 and T12, right two photos), 45% of the vocal folds had some contact when examined. These photographs were taken from the caudal (tracheal) end of the larynx and face cranially. Note also the shape of the trachea.

In 10 of 22 (45.5%) Texel larynges, the vocal folds were touching in the airway, in 20 out of 20 (100%) BFLs there was no contact between these structures (Figure 18). 

The BFL shows an elliptical shape, while the Texel trachea appears subjectively more rounded. In the Texel larynges, the vocal folds were more likely to contact in larynges with a wider angle of thyroid laminae.

### 3.5. Diagnostic Imaging

The CT images gave a clear view of the airway in the fixed larynges, but the MRI images provided a clear view of the airway and a clearer distinction between the laryngeal structures (CT and MRI images can be found in the Figure S1). T2 FSE and STIR sequences gave better differentiation between tissues due to bigger differences in signal intensity, while T1 GRE HR appears to show better resolution for anatomical detail. MRI also proved more useful for identifying pathology; imaging of TX1 found a small focal area of abscessation that was visualised on MRI and confirmed at gross dissection. 

### 3.6. Pathology

**Figure 19:**
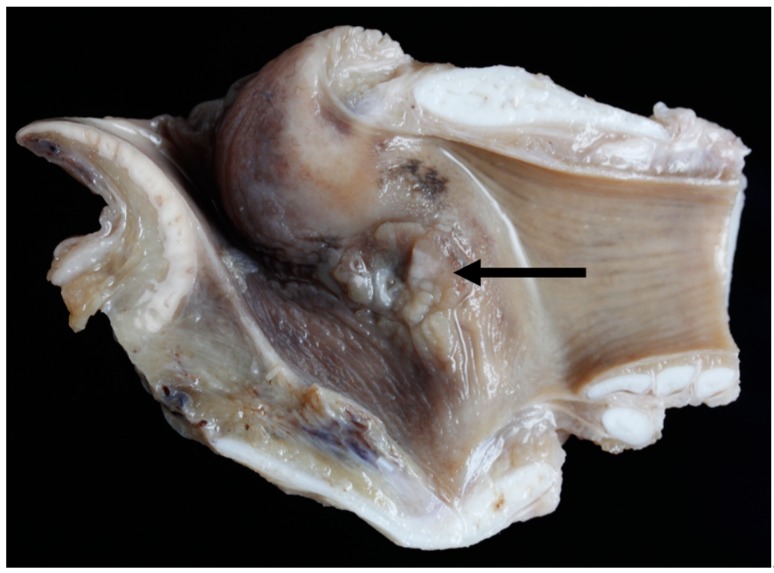
Right vocal cord moderate mucosal hyperplasia (arrow) [TX1].

**Figure 20:**
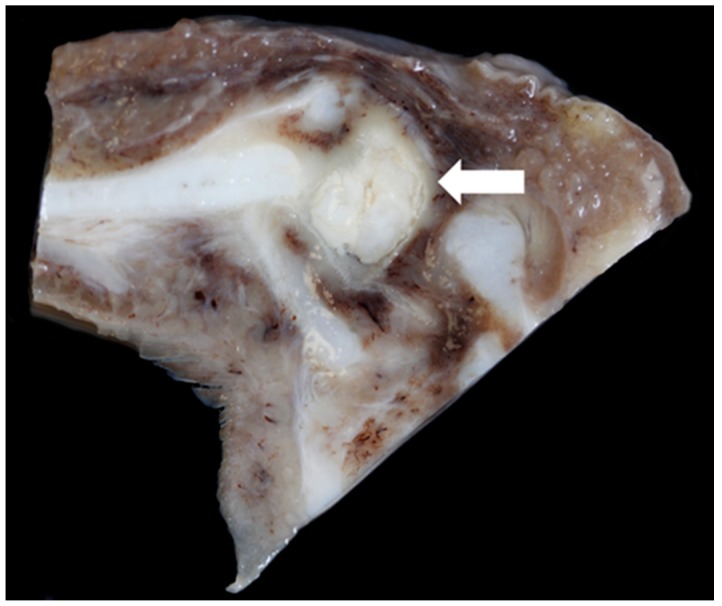
Medio-lateral slice through the left crico-arytenoid joint showing focally extensive abscessation (arrow) [TX9].

**Figure 21:**
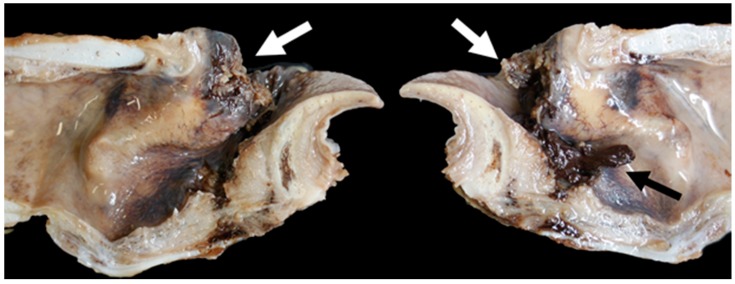
Massive bilateral necrosis of the corniculate process of the arytenoid cartilage (white arrows) with associated haemorrhage (black arrow) [TX13].

**Figure 22:**
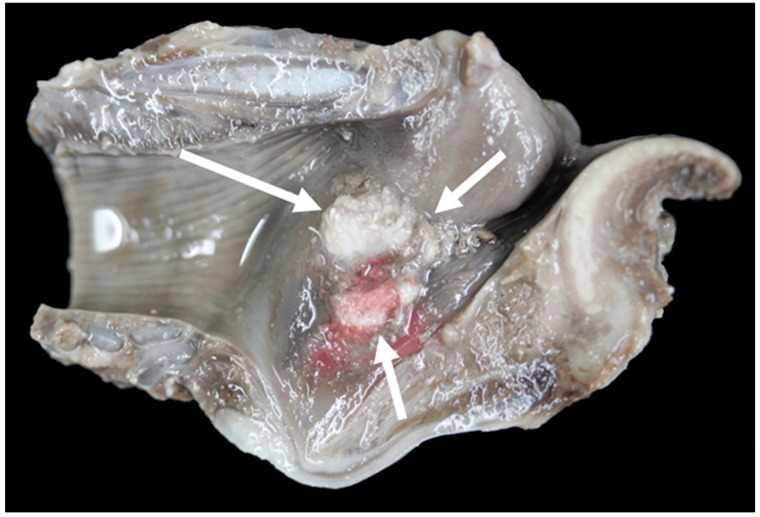
Severe unilateral vocal fold mucosal hyperplasia (arrows) (The red colouring is an artefact) [TX21].

Gross lesions were found in 52% (12/23) of the Texel rams compared to 10% (2/20) of the Bluefaced Leicesters. A description of the lesions observed is summarised in Table 3 and Figure 19, Figure 20, Figure 21 and Figure 22.

Compared to the control breed, the Texel sheep were significantly more likely to have laryngeal lesions (*p* = 0.01) and more likely to have contact between vocal folds (*p* < 0.001). It was not possible to predict lesions based on age.

## 4. Discussion

This study provides an initial insight into the anatomy of the Texel sheep larynx and suggests how it differs from a breed less prone to laryngeal chondritis. It has established a protocol for measuring and examining the larynx and highlighted the potential value of diagnostic imaging for use in future research projects. The authors are aware of no previous work investigating the normal anatomy of the sheep larynx. Zrunek et al. [12] studied the difference between the human and the ovine larynx, but the current study is the first to examine the sheep larynx in detail. This study has found that the overall shape, size and appearance of the larynx differs between individual sheep, and between breeds. 

The Bluefaced Leicester was chosen as a control in this study due to the ease of availability of carcases in the region of the UK where the larynges were collected. The two breeds, however, serve two different purposes in the UK sheep stratification system: BFL rams are used to produce North Country mules, a ewe used for breeding, whereas the Texel ram is used to produce lambs for meat. The Texel is, therefore, bred for its pronounced muscling and solid stance, while part of the breed description for the BFL is a good length of the neck [9,14]. The average weight of the two breeds of ram are similar; the Texel averages 120 kg (Texel Breed Society 2018, personal communication, 20 November) and the BFL ranges from 90 to 150 kg (Bluefaced Leicester Sheep Breeder’s Association 2018, personal communication, 16 November). Despite the Texel being a smaller animal in height and neck length (See Table 2), the larynx was not proportionately smaller in size compared to the BFL. Despite being of similar weights, the larynx of the Texel was also not a similar size to the Bluefaced Leicester. Although the larynx of the Texel is smaller in height and length, it has a funnel shape to the airway, and the epiglottis and arytenoid cartilages are disproportionately large for the supporting framework. This study has not found a significant difference in head length between breeds of sheep, which is the underlying cause of BOAS in brachycephalic dogs [8], but disproportion between supporting structures and soft tissue elements of anatomy play a large role in BOAS pathogenesis [15]. This study confirms that the Texel is more likely to have laryngeal lesions than the BFL, so disproportion of the Texel larynx may, therefore, play a part in predisposing the breed to laryngeal pathology. 

The disproportionate larynx and significantly smaller airway found in this study may also be a hindrance to animals with a high muscle mass. Texel sheep carry a large amount of muscle which makes their weight similar to that of the taller BFL, but muscle has a high oxygen demand. It is not known how the smaller larynx of the Texel affects the flow of air or the amount of air that is inspired and expired with each breath. The narrow airway of the Texel may, however, account for the increased respiratory rate often observed in these animals when compared to other breeds. The reduced airways found in this study should be taken into consideration at times of stress, hot weather, movement or handling when animals present with an increased respiratory rate or effort. 

In brachycephalic dogs, conformational factors have been shown to have a relationship with BOAS status [16]. Similarities could be drawn between brachycephalic dogs and breeds like the Texel, where some breeding lines have short, broad faces, short necks and narrower nostrils than other breeds. A hypoplastic trachea is a component of BOAS in dogs and is defined as a trachea with a markedly reduced lumen that does not vary in size during respiration [17,18,19]. The small size of the tracheal lumen found in this study in the Texel sheep supports a similarity to brachycephalic dogs, but the diameter during respiration of the live animal was not assessed here. The conformation of the Texel sheep also varies between different family lines. Some Texel sheep are bred for short necks, and it may be this that effectively ‘squashes’ the larynx to be shorter and wider. There are also anecdotal reports of longer necked animals having fewer laryngeal chondritis problems. The study was unable to demonstrate a correlation between the length of head or neck and the shape of the larynx, which may have been due to a small sample size, but it would be an important investigation point for further studies as it could prove a simple tool for predicting future laryngeal pathology. 

A range of pathology was observed in the collected larynges in this study. In the Bluefaced Leicester the lesions were mild and few while in the Texel sheep a wide range of lesions were observed, some of which were severe and may have caused the death of the animal. The duration of the lesions is unknown, but the prevalence of lesions in the Texel larynges suggests that laryngeal pathology, whether clinical or subclinical, could be more common than currently considered. Further longitudinal studies are required to correlate antemortem clinical findings with post mortem lesions. 

### 4.1. Imaging

MRI and CT scanning appear to be useful tools for assessing the anatomy of the formalin-fixed larynx and could be used for future research projects. Moving forward, a method to assess the anatomy of the larynx in the live sheep could be useful to prevent or predict laryngeal problems. In this study, a standing equine distal limb MRI scanner was used, more commonly used to scan the foot of the horse, and its use for live sheep would be practically impossible. Logistics and finances would likely exclude the use of a low field MRI scanner, as used in small animal and human hospitals, for routine examination of live sheep, although it would not be impossible. 

CT scanning is a diagnostic test that is often carried out in young breeding rams to assess muscle and fat carcase coverage using a mobile scanner in different geographical locations [20]. These animals are, however, sedated for examination, and the CT scanner only accommodates animals up to a certain weight. The effect of breathing in the live animal on laryngeal assessment, and the subsequent effect of sedation has also not been verified in sheep, but the larynx is routinely assessed in dogs by CT with 3D internal volume-rendered images of the larynx providing the most valuable information [18]. The value of CT scanning to assess laryngeal anatomy and/or predict future laryngeal problems in the live sheep would be valuable to investigate due to the current use of mobile CT Scanner Units in the industry making it a viable diagnostic test option. Although CT scanning could be a viable option for some groups of rams, it is not a viable diagnostic option when purchasing a ram at market, or for diagnosing an individual case of laryngeal chondritis on the farm. The use of ultrasound to assess the anatomy of the larynx was not investigated in this study but could be a viable option for on-farm investigations. The use of ultrasound as a tool to assess laryngeal anatomy, function and its use as a diagnostic tool for laryngeal lesions would also be a useful and realistic method to investigate.

### 4.2. Study Limitations

The protocol for examining and measuring the larynges evolved as the project progressed which resulted in some measurements not being collected on the early samples. All laryngeal measurements were taken on dead, fixed samples and it is unknown how this translates to the live animal, especially as the larynx is a functional structure. Comparison with the control suggests that the position of the arytenoid cartilages in the airway is genuinely more severe in the Texel than in the Bluefaced Leicester. 

Although an increased incidence of laryngeal chondritis is seen in male Texel sheep [4], the lesions can be found in all ages and in both sexes. This study focused on adult rams only because fallen stock of each breed could be reliably identified by appearance alone. Younger animals and ewes may have been more difficult to accurately select with no accompanying data or information about the animals. The effect of age on laryngeal anatomy or pathology was not possible to predict in this study, this may have been due to the sample size.

The clinical history of each animal sampled was unknown as they were collected at a fallen stock centre, it was, therefore, not possible to select animals that had been ‘clinically normal’, and some of the animals examined may have died from the lesions seen in larynx. It was not possible for the larynges to be blindly assessed as the same author collected the larynges and measured them, and individual larynges could be recognised throughout the process. 

## 5. Conclusions

This study confirms that there are differences in the Texel larynx, and the findings suggest that the anatomy could be having a detrimental effect on the health and function of the airway. For the incidence of laryngeal chondritis to be reduced, further understanding of the pathogenesis is required. More practically, a diagnostic test that allows susceptible animals to be recognised early, either before purchase or before breeding, could prevent losses occurring at crucial stages of the sheep year.

## Figures and Tables

**Table 1:** Description of carcase measurements.

Carcase Measurement	Points of Measurement
Head length	Caudal mandibular edge to cranial mandibular point of lower jaw
Neck circumference	Circumference of cranial neck, measured immediately caudal to ear base
Neck length	Cranial point of sternum to caudal mandible, measured along ventral neck
Height	Caudal ventral heel to withers
Girth	Circumference of girth

**Table 2:** A summary of the carcase and laryngeal measurements.

Anatomical Structure	Measurement	Texel		Blue-Faced Leicester		*p* Value
		*n*	Mean (mm)	*n*	Mean (mm)	
**Head and neck**	Head length	17	240	14	238	0.7
	Neck Circumference	17	495	14	404	<0.001
	Neck length	14	402	14	486	<0.001
	Height	17	742	14	843	<0.001
	Girth	16	1037	13	1034	<0.001
**Larynx length**	Anterior thyroid length	23	39.5	20	45.1	<0.001
	Dorsal thyroid length	23	55.8	20	65.1	<0.001
	Length of thyroid laminae	23	26.8	20	30.9	<0.001
	Main length of larynx	23	47.8	20	51.4	<0.001
	Split thyroid length	22	38.6	18	43.9	<0.001
	Length of cricoid cartilage	23	40.7	19	44.8	0.02
	Cricoid split length	22	34.4	18	38.8	0.02
	Anterior cricoid length	22	7.8	20	8.3	0.4
**Larynx width**	Maximum thyroid breadth	21	52.3	20	50.2	0.06
	Lower thyroid breadth	22	38.4	20	38.9	0.7
	Upper thyroid breadth	21	48.2	20	43.1	<0.001
**Larynx height**	Anteroposterior dimension	23	50.6	20	58	0.004
	Cricothyroid distance	20	43.3	18	51.5	<0.001
**Larynx angle**	Angle of thyroid cartilage	22	58.1°	20	50.4°	<0.001
**Airway**	Tracheal width	23	15.6	20	19	<0.001
	Tracheal height	23	19.2	19	23.9	<0.001
	Tracheal area	23	239 mm^2^	19	372 mm^2^	<0.01
**Arytenoid cartilage**	Corniculate process distance	21	15.7	19	16.8	0.23
	Depth of corniculate process	21	18.6	19	19.3	0.5
	Height of arytenoid cartilage	18	39.7	18	42.2	0.02
**Epiglottis**	Length of epiglottis	22	32.1	19	35.8	0.04

**Table 3:** Gross lesions observed in the larynges.

Larynx	Gross Lesion	Vocal Fold Contact
TX1	Moderate unilateral (right side) vocal fold mucosal hyperplasia	Yes
TX4	Moderate haemorrhage in right dorsal cricoarytenoid muscle, mild bilateral vocal fold mucosal erosion and hyperplasia	Yes
TX6	Mild bilateral vocal fold mucosal hyperplasia	No
TX7	Mild unilateral vocal fold mucosal hyperplasia	No
TX8	Mild vocal fold mucosal erosion	No
TX9	Unilateral (left side) mild vocal fold mucosal hyperplasia, right-sided mild vocal fold mucosa flattening, bilateral severe suppurative cricoarytenoid osteoarthritis	Yes
TX11	Bilateral, moderate vocal fold mucosal hyperplasia	No
TX12	Bilateral, mild, vocal fold mucosal ulceration	Yes
TX13	Massive bilateral necrosis of the corniculate process of the arytenoid cartilages with haemorrhage	No
TX16	Bilateral moderate vocal fold mucosal hyperplasia	Yes
TX17	Bilateral moderate vocal fold mucosal hyperplasia	Yes
TX21	Severe bilateral (left sided) vocal fold mucosal hyperplasia	Yes
BL5	Mild unilateral vocal fold mucosal hyperplasia	No
BL20	Mild unilateral vocal fold mucosal hyperplasia	No

## Supplementary Materials

The following are available online at https://www.mdpi.com/2306-7381/6/1/21/s1, Supplementary Figure S1: CT and MRI images of the fixed larynges.
