# Supplementary material for: Anatomy and Pathology of the Texel Sheep Larynx
Source: Vet Sci. 2019 Feb 27;6(1):21. doi: 10.3390/vetsci6010021 (PMC6466285; doi:10.3390/vetsci6010021)
Supplement: Supplementary file 1 [file vetsci-06-00021-s001.pdf]

Article

Supplementary Material: Anatomy and Pathology of the Texel Sheep Larynx

Katie Waine [1,*], Ben Strugnell [2], John Remnant [1], Fiona Lovatt [1], Martin Green [1], Hannah Rideout [3], Elizabeth Genever [4] and Kerstin Baiker [1]

[1] School of Veterinary Medicine and Science, University of Nottingham, LE12 5RD Leicestershire, UK; John.remnant@nottingham.ac.uk (J.R.); fiona.lovatt@nottingham.ac.uk (F.L.); martin.green@nottingham.ac.uk (M.G.); kerstin.baiker@nottingham.ac.uk (K.B.)
[2] Farm Post Mortems Ltd, Hamsterley House, Hamsterley, Bishop Auckland, DL13 3QF Durham, UK; benstrugnell@aol.com
[3] Harper Adams University, Newport, TF10 8NB Shropshire, UK; rideouth10@gmail.com
[4] Liz Genever sheep and beef consultancy, Bramley Cottage, Uffington, Stamford, PE9 4SX Lincolnshire, UK; kerstin.baiker@nottingham.ac.uk
* Correspondence: Katie.waine1@nottingham.ac.uk

Received: 23 January 2019; Accepted: 20 February 2019; Published: 27February 2019

Figure S1. CT and MRI images of the fixed larynges.

CT images

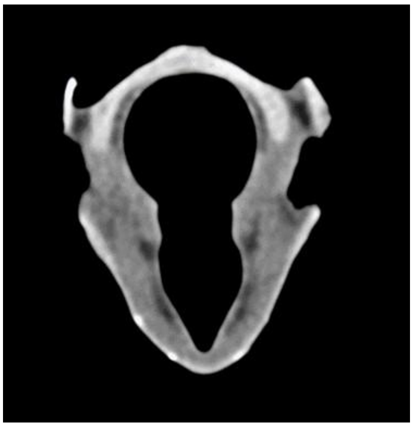

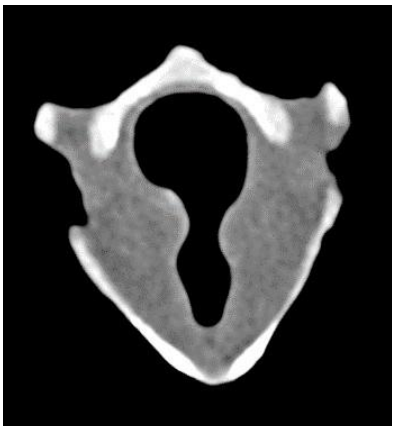

CT images at the narrowest point of the glottis: BL3 (left) and TX2 (right). The central black area in the middle of the greyish structure (laryngeal cartilages and soft tissue) is the airway. The bright white structures are the laryngeal cartilages and the grey areas are the soft tissue.

MRI images

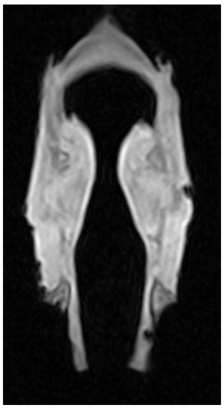

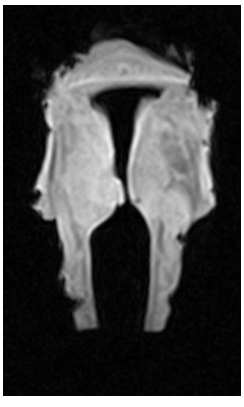

T1W 3D HR Frontal slices (BL8 left, TX1 right)

veterinary sciences

MDPI

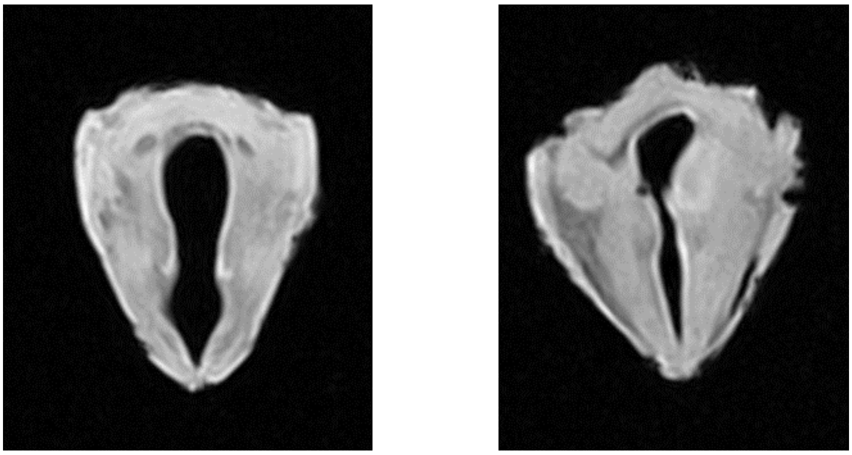

T1W 3D HR Transverse slices (BL8 left, TX1 left)

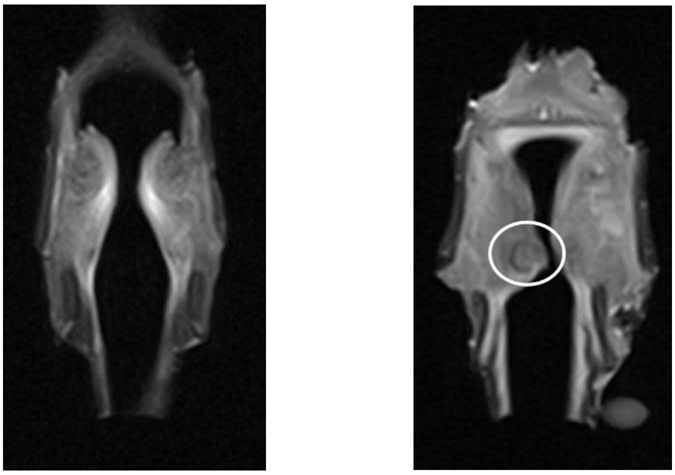

FSE HR Frontal slices (BL8 left, TX1 right) showing focally extensive abscess formation in TX1 (white circle).

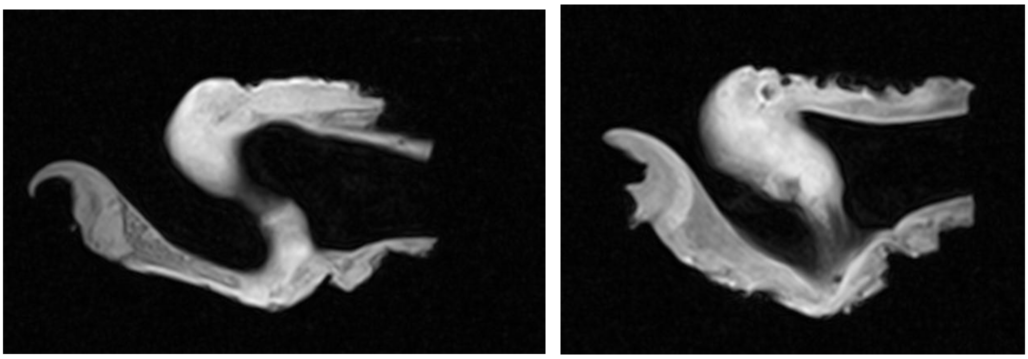

T1W 3D HR Sagittal plane (BL8 left, TX1 right)
